# An imaging system integrating optical coherence tomography and interferometry for in vivo measurement of the thickness and dynamics of the tear film

**DOI:** 10.1186/s12938-018-0597-y

**Published:** 2018-11-01

**Authors:** Yuqiang Bai, William Ngo, Boyu Gu, Yuhua Zhang, Jason J. Nichols

**Affiliations:** 1 School of Optometry, University of Alabama at Birmingham, Birmingham, AL 35233 USA; 2 Department of Ophthalmology, University of Alabama at Birmingham, Birmingham, AL 35233 USA

**Keywords:** Interferometry, Optical coherence tomography, Tear film, Lipid layer

## Abstract

**Background:**

The outermost layer of the tear film consists of a thin lipid layer (LL). The lipid layer serves as a barrier against evaporation of the aqueous component of the tear film. The ability to simultaneously image both the lipid layer thickness and overall tear film thickness is novel, and will help further understandings of mechanisms of how the lipid layer assembles and interacts with the full tear film thickness.

**Methods:**

We developed a system that combines simultaneous optical coherence tomography (OCT) and thickness dependent fringes (TDF) interferometry for in vivo imaging of the tear film. The OCT possesses an axial resolution of 1.38 µm in tear film, providing an accurate measurement of the thickness of the overall tear film. The TDF can detect a minimal change of approximately 15 nm in LL thickness. In addition, the spatial resolution of TDF images in x–y plane is 5 µm.

**Results:**

The effect of instilling artificial tears on the PCTF and PLTF was examined. In both contact lens and non-contact lens wear, it could be observed from the OCT results that instillation of artificial tears increased the thickness of the overall tear film immediately, followed by a gradual reduction thereafter. These findings were consistent with other studies. However, unlike those previous reports, the thickness of the LL in this study was quantified simultaneously with the TDF subsystem. The results showed that bulking agents such as these artificial tears were not necessarily intended to increase the LL thickness. Immediately after instillation of artificial tears, the PCTF increased from 4.4 ± 0.97 to 20.3 ± 3.6 µm. The PCTF then decreased to 8.8 ± 2.1 µm at 4 min post-instillation. The thicknesses of the LL were 62.4 ± 14.5 nm, 48.7 ± 5.3 nm, and 55.2 ± 9.8 nm at pre-drop instillation, post-drop instillation, and 4-min post-drop instillation, respectively.

**Conclusions:**

In this work, we have described a novel imaging system that integrated OCT and TDF imaging techniques, which may facilitate the study of many physiological and clinical aspects of the tear film.

## Background

The fluid over the ocular surface is known as the precorneal tear film (PCTF). The PCTF is approximately 3–5 µm thick and it provides lubrication for the cornea, as well as a smooth optical interface for light to enter the eye and pass to the retina [1]. The outermost layer of the PCTF is known as the lipid layer (LL), a thin film composed of polar and nonpolar lipids. The LL rests anterior to the tear film aqueous, which is composed of proteins, electrolytes, and a gradient of soluble and membrane bound mucins (at the corneal surface). The tear volume on the ocular surface closely relates to ocular surface disease [2–5]. Thus, precise measurement of the thickness and dynamic visualization of the PCTF allow for deeper understanding of the tear film’s structural and functional roles in ocular health and associated clinical ocular surface-related conditions [3, 6, 7].

The LL comprises a very small fraction of the overall PCTF thickness (~ 40–100 nm in healthy humans). It serves to maintain the stability of the PCTF and is an important barrier for reducing the evaporation of the aqueous phase [6, 8–11]. The measurement of the LL thickness over the ocular surface enables characterization of a number of physical parameters such as its spread after a blink, and the differences of LL thickness/thinning rates among different regions of the ocular surface [8, 10, 12–15]. It is valuable to be able to evaluate the LL and PCTF thickness simultaneously, as it will allow us to investigate the relationship between the LL and PCTF evaporation rate, and will allow us study the distribution or characteristics of the LL and its relationship to the stability of the PCTF. It is also valuable to understand mechanisms of LL formation and rupture, which can be studied in vivo using these imaging techniques while simultaneously understanding the impact of lipid layer formation and rupture on the PCTF.

Tear film thickness measurements and dynamic visualization of the LL and overall tear film have been characterized by different optical approaches separately, such as optical coherence tomography (OCT), confocal microscopy, wavelength-dependent fringes (WDF), thickness-dependent fringes (TDF) and angle-dependent fringes (ADF) [7, 16–20]. These different methods have shown advantages and disadvantages in study of tear film structure and dynamics.

Modern ultra-high resolution OCT has been demonstrated for evaluating the thickness of overall PCTF [21–23]. With the scheme of 2-axis scanning and fast image acquisition speed, OCT has the potential to measure the PCTF thickness at various depths across the ocular surface. However, the axial resolution of the OCT is limited down to 1 μm. In contrast, traditional interferometric methods have better resolution, but they are limited to measuring a single, small spot ~ 30-µm diameter on the ocular surface. With thickness dependent fringes (TDF) interferometry, the tear film LL thickness can been quantified based on the interference patterns that result from light reflected from the air-lipid and lipid-aqueous interface [24–26]. For a given thickness at one point, the intensity of the reflected light is a sinusoidal function of the wave number, meaning the dominant reflected color is dependent on the thickness of the film. With a relatively large field of view of the spatial distribution of the LL (a circular area 2–8 mm in diameter), sequential interference images can be used to quantify the thickness, spread, distribution, and movement of the LL between blinks.

The purpose of this work was to demonstrate the development of an imaging system that integrates OCT and TDF interferometry. Using these two combined approaches, the full PCTF thickness and LL thickness are measured simultaneously.

## Methods

### System design

**Fig. 1:**
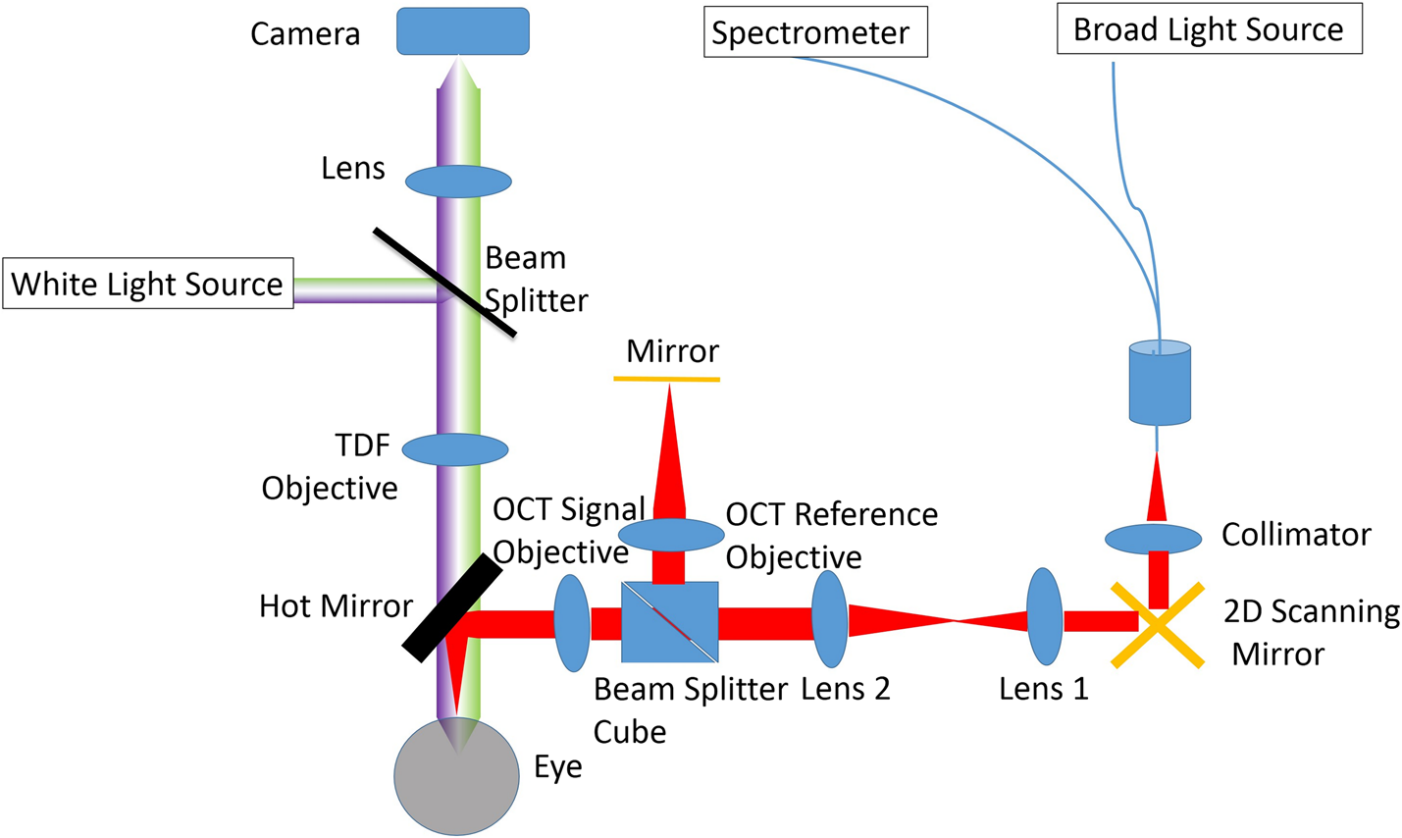
Schematic setup of the integrated TDF and OCT system. Light path of the TDF system is colored in green-purple, while the path of the OCT in red. The assembly of Lens 1 and 2 works as a telescope in OCT path

Figure 1 shows the schematic of the integrated OCT and TDF system. The laser light source for the OCT is a broadband superluminescent diode (T850-HP, Superlumdiodes, Russia) with a central wavelength of 840 nm and full width half maximum (FWHM) of 175 nm. In conventional fiber-based OCT systems, a 2 × 2 fiber coupler delivers the light in separate signal and reference arms. This system design results in a significant mismatch of group velocity dispersion (GVD) and polarization between the signal and sample arms, which decreases the axial resolution of the system [27]. To maintain the system in a compact size while minimizing the mismatch between the sample arm and the reference arm, the current OCT system is set up as a hybrid between a fiber and free-space type. Light emitting from the source is split by a 1 × 2 fiber coupler, with which 50% of the light is transmitted as the imaging probe. The light probe is collimated (RC08FC-P01, Thorlabs, Newton, New Jersey) and scanned by the galvanometer scanner. A dual-lens telescope is positioned after the scanner in a way that the scanning mirrors and back focal aperture (BFA) of the objective are in conjugate planes [28–30]. The scanning mirrors create a pivot point at the BFA where the beam is stationary in a 2D plane. Thus, the arrangement allows for large scanning angles while minimizing lens aberrations. The focal length of the first and second lens in the telescope is 50 mm and 75 mm, respectively.

A 90/10 cube beam splitter is positioned right after the second lens of the telescope, which separates the probe into the signal and reference arm. The light in the signal arm is focused onto the ocular surface by the objective lens. The reflected light from the ocular surface is combined with the returning beam of the reference arm at the cube beam splitter. The combined light transmits inversely along the ingoing path to the 1 × 2 fiber coupler, through which 50% of the light is delivered to one custom-made spectrometer. Under the current configuration, light from the signal and reference arms is split and interferes in free space, and is then collected by the same physical path of fiber. Thus, GVD and polarization mismatch between the reference and the sample arm are minimized, while a compact size is maintained. If the cube beam splitter is placed behind the scanner, the reference arm and signal arm is split and an extra telescope would be needed in the reference arm. Although the extra telescope can be identical to the one in the signal arm, they will not be exactly the same and will increase the mismatch of dispersion and GVD between the signal and reference arms. In addition, the insertion of another telescope will complicate and increase the system size. One disadvantage of this arrangement is that the probe scans in both reference and sample arms, and the back coupling of the reflected light from the reference arm varies during scanning. To address this issue, the light in the signal arm is blocked and the reflected light from the reference arm is recorded point-by-point along the scanning path. Then, the reference signal is subtracted from the interference signal point-by-point prior to the Fourier transform. The data processing algorithm of the current system follows the same method of conventional OCT systems and any possible noise is avoided.

The system uses two identical telecentric scan lenses (LSM04-BB; Thorlabs, Newton, New Jersey) as objectives in the sample and reference arm to minimize the dispersion mismatch between them and minimize the distortion of the imaging. The scan lens has a focal length of 54 mm and a transverse resolution of 20 µm on the sample. The custom-made spectrometer follows the previous design [31, 32]. In short, the spectrometer consists of a collimating lens with a focal length of 75 mm (Edmund Optics, Barrington, New Jersey), a 1800 lines/mm volume holography transmission grating (Wasatch Photonics, Logan, Utah), an assembly of double lenses with an effective focal length of 150 mm, and a high-speed line array CCD camera (spL4096-140k, Basler, Highland, Illinois). The camera has 4096 pixels and maximum readout rate of 140 kHz. The data processing algorithm, control, and display software are developed using LabVIEW (National Instruments, Austin, Texas).”

The axial resolution of an OCT system is determined by the coherence length of the light source. If the spectrum of the light source has a Gaussian profile with central wavelength $\lambda_{0}$ and a bandwidth $\Delta \lambda$ as the FWHM, the axial resolution of an OCT system, δz, can be expressed as:

1 $$\updelta{\text{z}} = \frac{{2ln2\lambda_{0}^{2} }}{\pi \Delta \lambda }$$

**Fig. 2:**
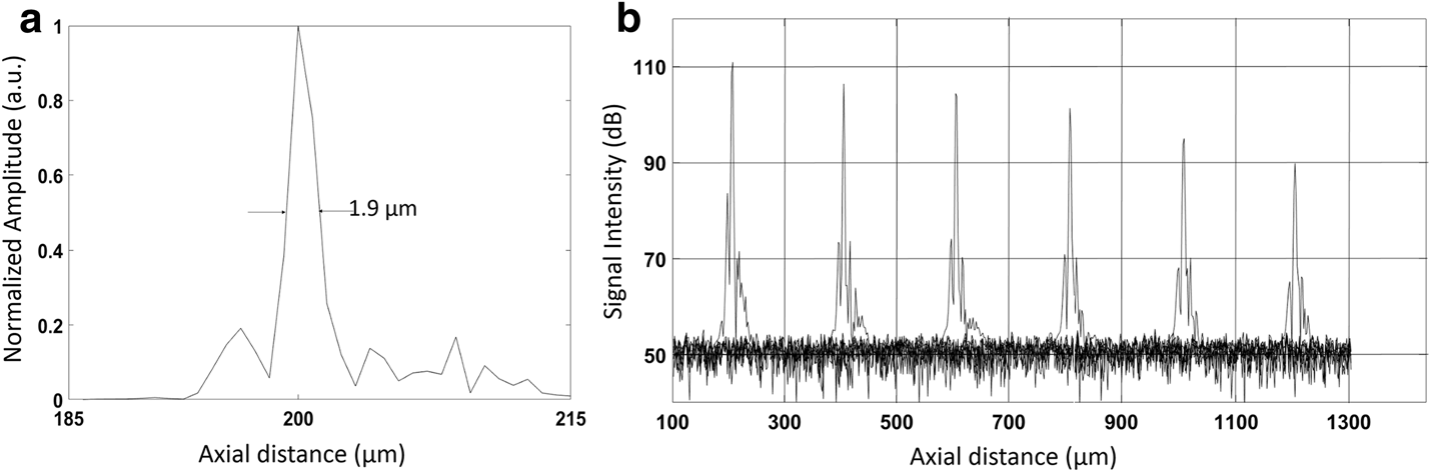
**a** The axial PSF measured from a mirror at path difference of 200 µm. The FWHM of the axial PSF at this position is 1.9 µm in air, corresponding to 1.38 µm in corneal tissue. **b** Sensitivity fall-off of the system evaluated by measuring the axial PSF at different depths

The FWHM of the PSF (point spread function) of the OCT system was measured to obtain the axial resolution by using a mirror as a sample. As shown in Fig. 2a, the FWHM of the PSF is 1.9 µm at the axial distance of 200 µm, corresponding to an axial resolution of 1.38 µm in tear film (n = 1.376). The effective ranging depth is defined as the imaging depth at which the sensitivity decays 6 dB from the peak value. The depth range of the current system is 600 μm. The sensitivity of the OCT system along the axial distance is displayed in Fig. 2b.

**Fig. 3:**
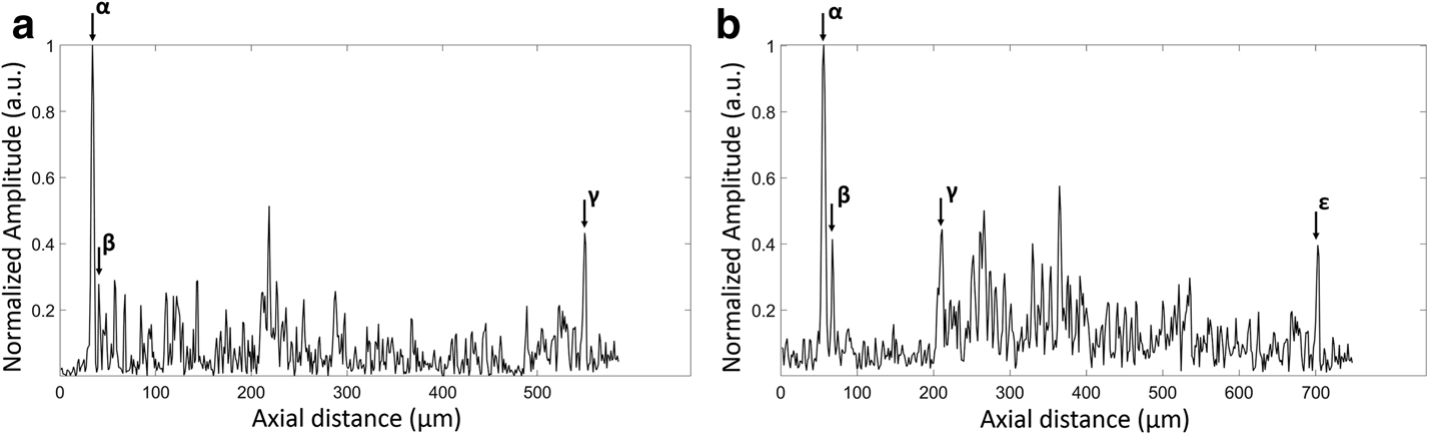
Representative OCT intensity profiles for the measurement of the tear film thickness. **a** A-scan of the precorneal tear film (PCTF). α, the anterior surface of the PCTF; β, the posterior surface of the PCTF; γ, the posterior surface of the cornea. **b** A-scan of the prelens tear film (PLTF). α, the anterior surface of the PLTF; β, the posterior surface of the PLTF or the anterior surface of the contact lens; γ, the posterior surface of the contact lens; ε, the posterior surface of the cornea. The thickness of tear film layer is calculated by determining the distance between peaks α and β

The power of the incident light focused onto the mirror is 0.8 mW. During in vivo tear film measurements, the incident power output is modulated between 0.4 mW to 0.8 mW to avoid the saturation of OCT signal at the central cornea. A customized software combining numerical dispersion compensation and spectral shaping is developed to provide an axial resolution sufficient for resolving the boundaries of the tear film [33]. After a Fourier transform of the interference signal, a depth-resolved reflectivity profile is obtained from each A-scan using MATLAB (MathWorks, Natick, Massachusetts) and the thickness of the tear film is interpreted as the distance between the signal peaks at the air-tear film and tear film-cornea interfaces as shown in Fig. 3. To increase the point density of each A-line, improve the quality of the linear interpolation, and suppress the sidelobes of PSF, the detected spectrum is zero padded to a length of 8192 pixels before Fourier transform [34–36]. The average tear film thickness is calculated as the mean value over B-scan. All axial distance values for the tear film obtained with OCT are divided by the average group refractive index for the tear film of 1.376 to get geometrical distances. After that, ImageJ (National Institutes of Health, Bethesda, Maryland), is used to enhance the cross-sectional images by the adjustment of brightness and contrast.

TDF interferometry system has been previously described elsewhere, although its combination and co-registration with a high resolution OCT is novel [8, 13, 37]. In short, a Quartz Tungsten-Halogen Lamp (QTH10, Thorlabs, New Jersey) is used as the illuminating source, which is reflected from a glass plate beam splitter and imaged at the center of curvature of the cornea. The light falling on the cornea at normal incidence is reflected straight back along its incident path. The reflected light is focused and forms an image of the ocular surface via a video camera (avA1000-100gc, Basler, Highland, Illinois). Uncompressed video recordings are made at 30 frames per second with an exposure duration of 30 ms. Long-pass and short-pass filters are combined together to attenuate wavelengths outside of the visible range (400–700 nm). The TDF system has a circular field of view of 3 mm and a spatial resolution of 5 μm in x–y plane. The LL thickness is quantified and graded by identifying the dominant colors in the interference patterns [12, 38]. White reflectance corresponds to thicknesses below 30 nm, grey up to 60 nm, yellow up to 90 nm, brown up to 135 nm, and blue up to 180 nm. In this study, the algorithm to measure LL thickness quantitatively is developed from our previous work [13, 26]. Briefly, the ratio of the reflected intensities of the different wavelengths is the function of LL thickness and can be simulated by thin film reflectometry [13]. The video camera is configured in the RGB model. The ratios among R, G, and B values from the measurement are compared and fitted with those from the simulation to obtain lipid layer thickness values.

A hot mirror is applied in the system to combine the light sources of the OCT and TDF and to separate the return signals for the two imaging modules.

### Calibration of OCT-TDF and co-registration of images

**Fig. 4:**
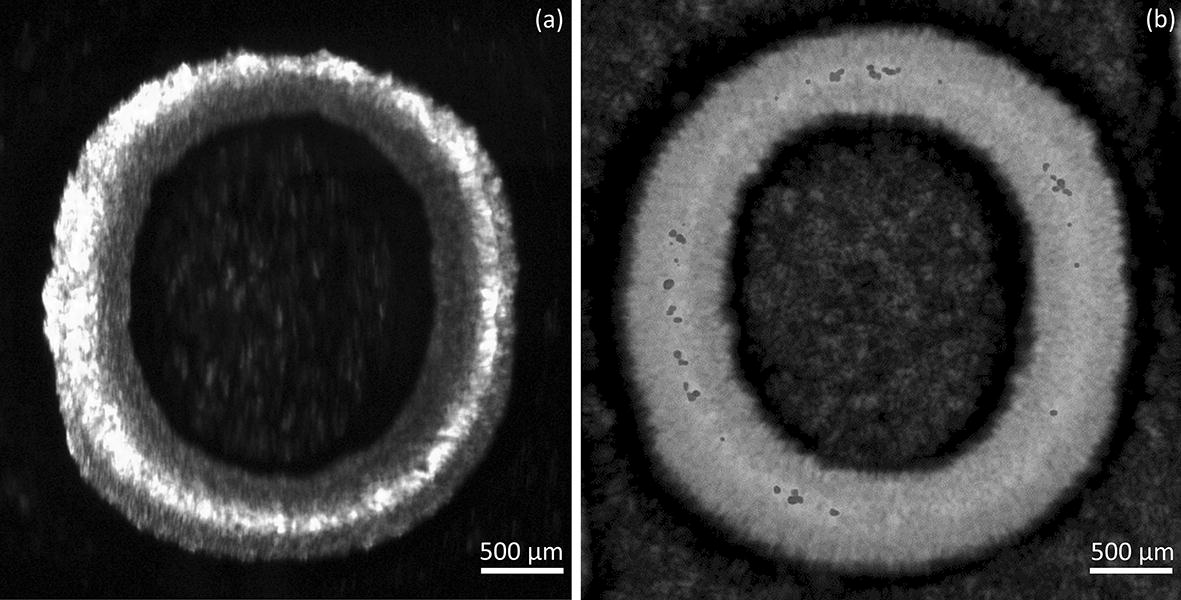
Co-registration image of **a** TDF and **b** OCT of an embossed letter “O”. The TDF and OCT system was calibrated with the internal edge of the letter

The co-registration of the combined OCT and TDF system was validated by constructing OCT en-face images and comparing them to the TDF system. Multiple C-scans with a size of 3 × 3×1.5 mm (256 × 256 × 520 pixels) were generated by ImageJ by using a stack of OCT B-scans collected at different lateral positions from the sample. The OCT en-face images were then generated by extracting and summing signals at a constant depth (1.5 mm) of the C-scan. Orientation of the CCD camera for TDF and scanning voltage for OCT was adjusted for the purpose of co-registration. Figure 4 shows the images from the OCT and TDF. In this example, the embossed letter “O” from a ruler is displayed at the same position in both images, demonstrating the spatial co-registration of the two functional modules in a single platform.

**Fig. 5:**
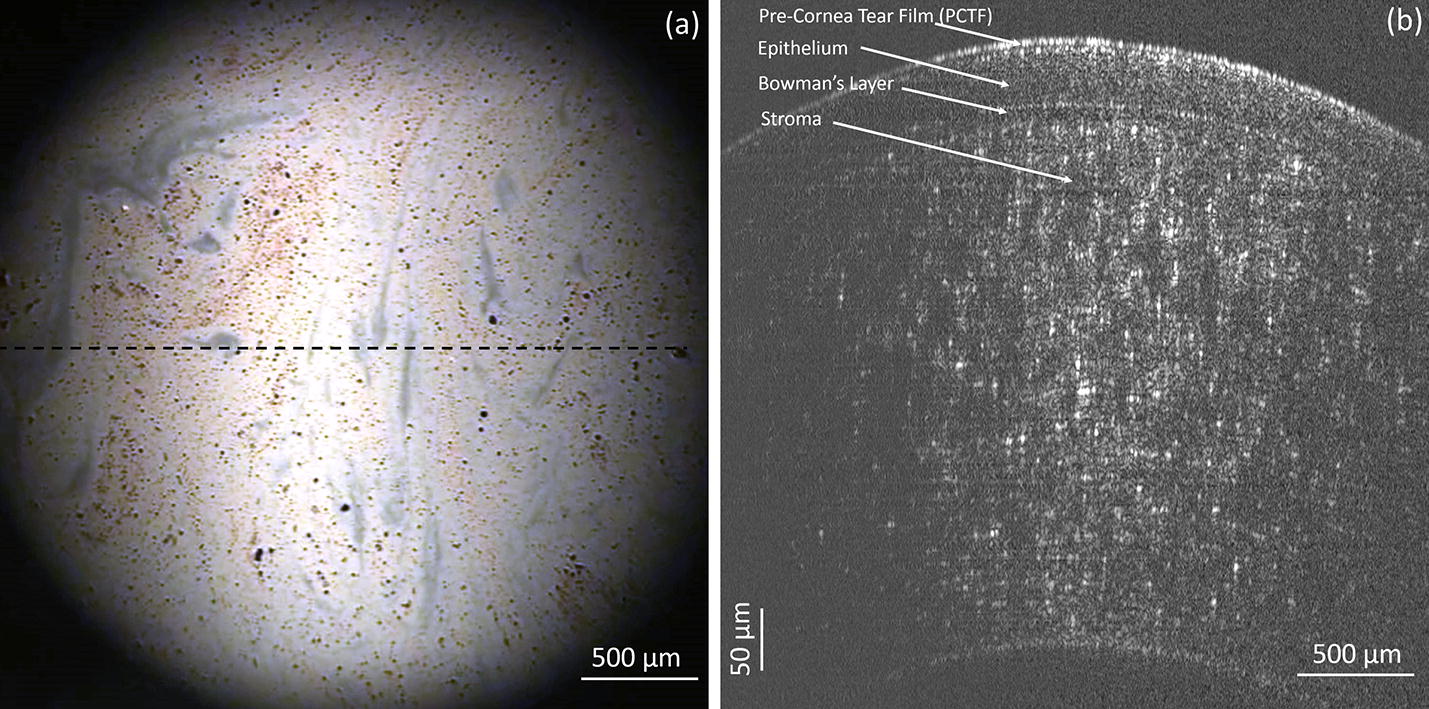
Co-registration image of **a** TDF and **b** OCT of a precorneal tear film in vivo. The dashed line in Fig. 5a indicates the position of the OCT B-scan in **b**. The thickness of the lipid layer in **a** and that of tear film in **b** is 5.8 ± 0.7 µm and 77.3 ± 15.2 nm, respectively

This system was adapted to image human subjects by adding a chinrest and forehead rest to stabilize the head of a human subject. This chin and forehead rest can be adjusted to move along the 3 cardinal axes of movement to align the human subject’s eye with the optics of the system. Figure 5 shows an image of a human pre-corneal tear film acquired by the OCT and TDF in vivo. The black dotted line on the TDF (Fig. 5a) indicates the location of the OCT B-scan on the horizontal meridian of the central cornea (Fig. 5b). As shown in Fig. 5a, the thickness variation of the LL can be observed as varying color patterns due to interference fringes. The average LL thickness over the entire image is 77.3 ± 15.2 nm. For the OCT image in Fig. 5b, the PCTF can be clearly visualized along with the corneal epithelium, Bowman’s layer, and anterior stroma. The average thickness of PCTF in the image is 5.8 ± 0.7 µm.

### Measuring the effect of artificial tears on the PCTF and PLTF thickness

Over the counter artificial tears (Refresh Liquigel with mid-viscosity (1.0% CMC (carboxy methylcellulose)); Allergan, Irvine, CA) were instilled onto the eye to show the impact of adding a bulking agent to the PCTF in both a normal individual and in a soft contact lens wearer (to measure the pre-lens tear film, PLTF). After capturing baseline images using the OCT/TDF system, the tear film was imaged again immediately after instilling one drop (~ 50 µl) of artificial tears and then again at 4 min post-instillation. While the TDF imaged the LL over the central 3 mm of the cornea, the OCT scanned the middle of the same region simultaneously.

### Measuring the PCTF thinning rate

The thinning rate of the PCTF was demonstrated on the same healthy individual. The subject was asked to blink three times before being asked to keep the eyelids open for 20 s. Measurement of PCTF thinning rates started at 2.5 s after the final blink to avoid transients due to upward drift of the PCTF [39]. Due to the limit of the acquisition speed of the spectrometer, the OCT has to sacrifice sampling density to synchronize with the TDF speed. While the line scan camera in the current system enables a maximum readout rate of 140 kHz. The OCT system was operated at at 32.8 kHz to have sufficient exposure duration. To synchronize the acquisition speed of the OCT and TDF, the time to complete a coarse OCT C-scan was set to equal the exposure time for each TDF frame. Under the current acquisition rate, the OCT scanned 8 horizontal lines (each comprising of 128 A-lines) of the PCTF, evenly spaced along the vertical direction within the 3 mm diameter zone. With a measurement time of approximately 30 ms per C-scan, both the OCT and TDF data were simultaneously acquired at a rate of 32 frames per second. The PCTF thinning rate for the 1st, 5th, and 8th horizontal lines, representing the thinning rate for the superior, central, and inferior 3 mm zone of the central cornea, respectively, were calculated by analyzing the series of OCT images using ImageJ. Regression lines were fitted to thickness-versus-time data starting at 2.5 s after the blink, and ending at 20 s [39].

All the experiments were conducted in a dimmed room with controlled temperature (23–25 °C) and humidity (30–50%).

## Result

### The effect of artificial tears on the PCTF and PLTF thickness

**Fig. 6:**
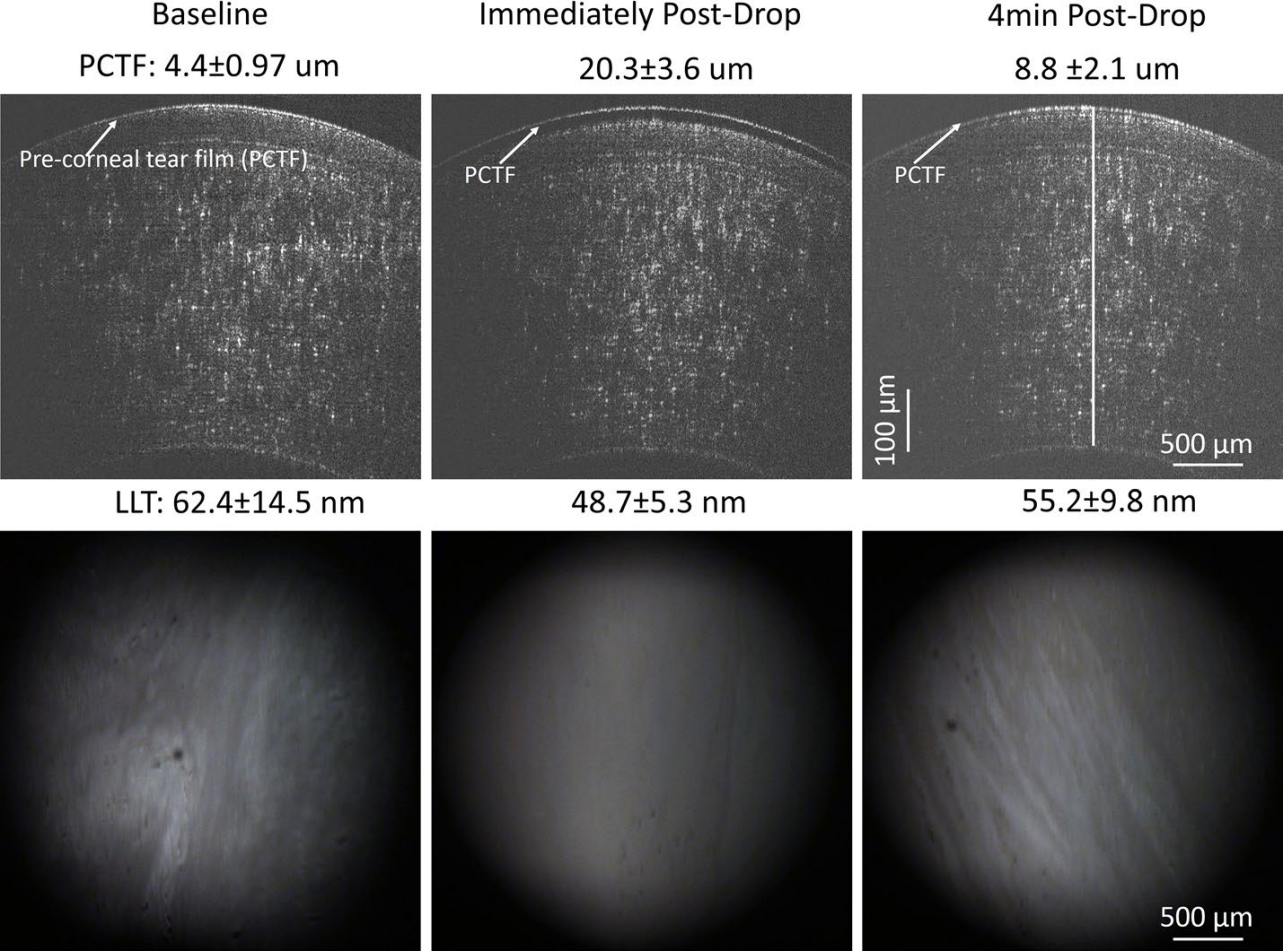
Images of TDF (lower) and OCT (upper) of the PCTF with artificial tears

**Fig. 7:**
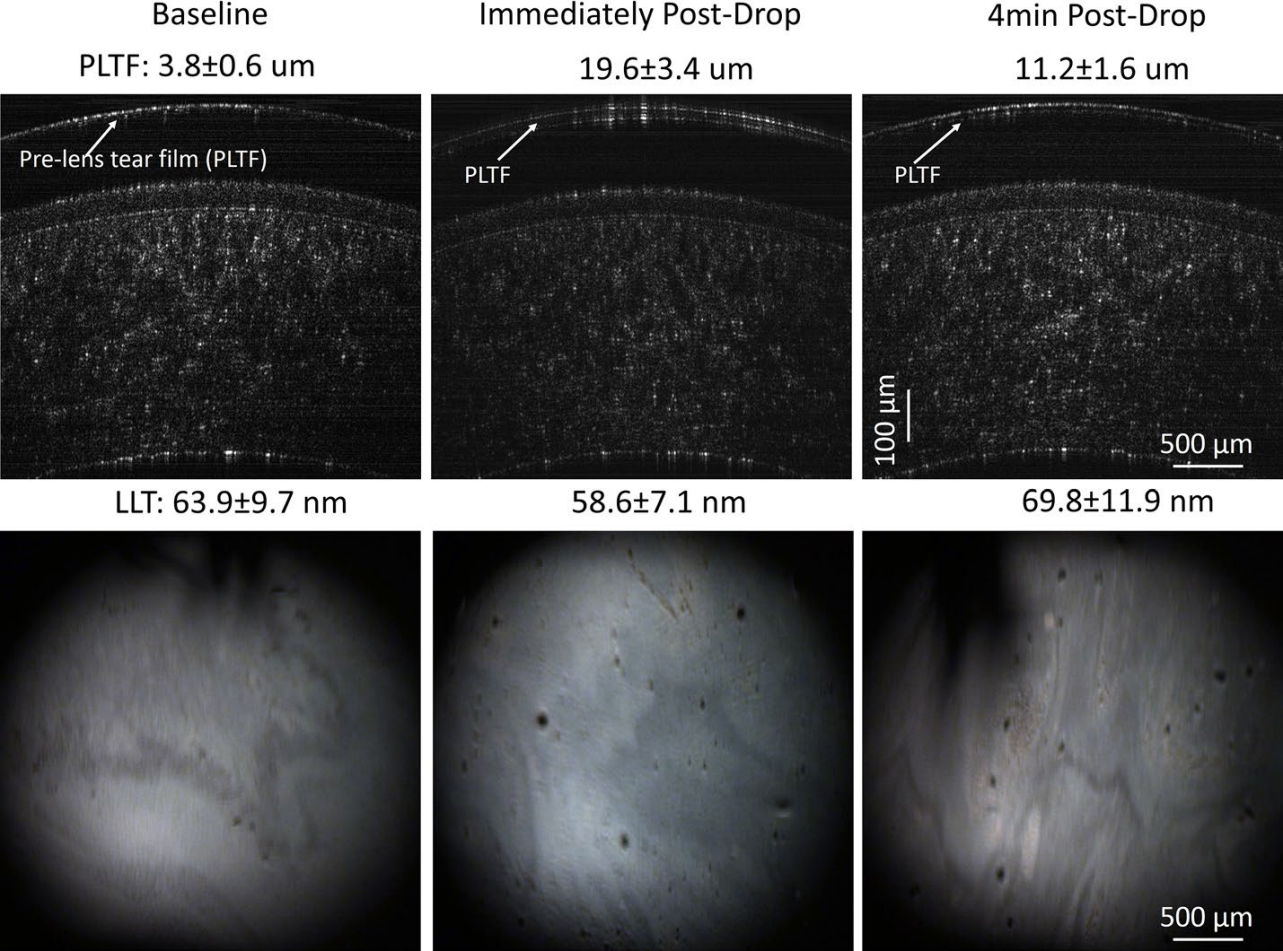
Images of TDF (lower) and OCT (upper) of the PLTF with artificial tears in a contact lens wearer in vivo

The PCTF and PLTF were imaged before and immediately after instilling one drop of artificial tears (Figs. 6, 7). Immediately after instillation of artificial tears, the PCTF increased from 4.4 ± 0.97 µm to 20.3 ± 3.6 µm. The PCTF then decreased to 8.8 ± 2.1 µm at 4 min post-instillation. The thicknesses of the LL were 62.4 ± 14.5 nm, 48.7 ± 5.3 nm, and 55.2 ± 9.8 nm at pre-drop instillation, post-drop instillation, and 4-min post-drop instillation, respectively. In the contact lens wearer, the PLTF increased from 3.8 ± 0.6 µm to 19.6 ± 3.4 µm immediately after instillation, and then decreased to 11.2 ± 1.6 µm at 4 min post-installation. In the contact lens wearer, the thicknesses of the LL of the PLTF were 63.9 ± 9.7 nm, 58.6 ± 7.1 nm, and 69.8 ± 11.9 nm at pre-drop instillation, post-drop instillation, and at 4 min, respectively.

### The thinning rate of the PCTF

**Fig. 8:**
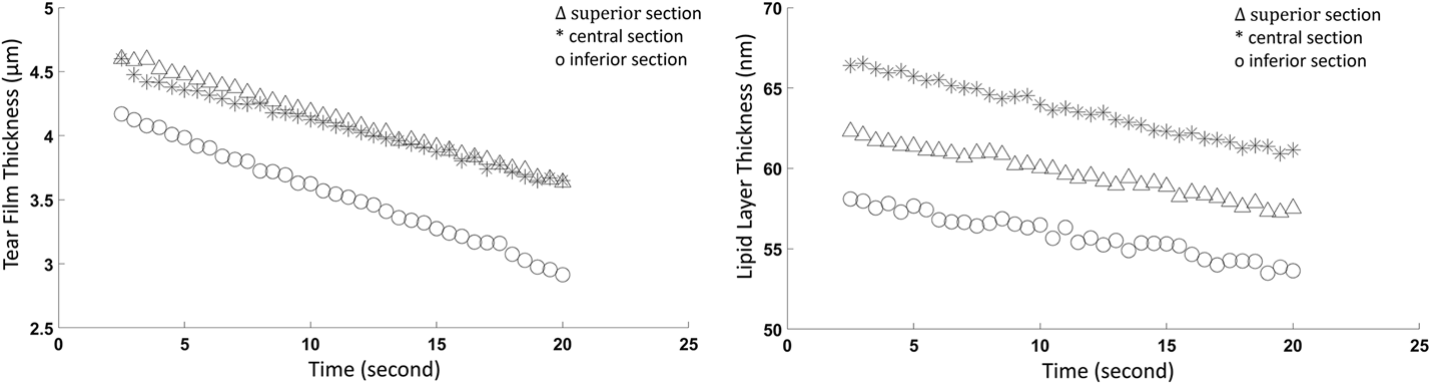
The thinning rate of the PCTF around the central region of cornea. The time scale started at 2.5 s after the blink, the OCT scanned three transversal sections (superior, central, and inferior) within the 3 mm diameter zone of the central PCTF over a course of 20 s (left). Simultaneously, the TDF measured the lipid layer thickness of the entire 3 mm diameter zone over a course of 20 s (right)

The thinning rate of the PCTF was demonstrated in the normal individual over 20 s as presented in Fig. 8. The initial PCTF thicknesses were 4.6 μm, 4.6 μm, and 4.1 μm, and the PCTF thinning rates ([95% CI], R^2^) were 3.4 µm/min ([3.3, 3.4], R^2^ = 0.998), 3.1 µm/min ([2.9, 3.1], R^2^ = 0.993), and 4.2 µm/min ([4.1, 4.2], R^2^ = 0.998) for the superior, central, and inferior PCTF areas, respectively. The initial lipid layer thicknesses were 58.1 nm, 66.4 nm, and 62.3 nm, and the LL thinning rates ([95% CI], R^2^) were 15.3 nm/min ([14.4, 16.7], R^2^ = 0.912), 18.7 nm/min ([16.2, 19.3], R^2^ = 0.913), and 17.4 nm/min ([15.9, 18.4], R^2^ = 0.965) for the superior, central, and inferior PCTF sections, respectively.

## Discussion

Precise measurement of the thickness and dynamic visualization of the PCTF allow for deeper understanding of the tear film’s structural and functional roles in ocular health and associated clinical ocular surface-related conditions. For instance, tear film breakup corresponds to a dry spot or very thin PCTF over the cornea. TDF technique has been widely used to investigate the breakup of the tear film by charactering the dynamics of LLT along the process [8, 40]. However, as the breakup is the final result of the thinning of the tear film, the thickness dynamics of the PCTF, crucial to fully understand the breakup events, are still unknown yet. By combining of TDF with OCT together, it provide a potential means to accurately characterize the thickness of PCTF and the LLT simultaneously in the events of tear film breakup. Data collected using this system may provide new insight into understand the mechanism of tear film breakup.

Various methods of imaging the thickness of the PLTF or PCTF, and specifically the LL, have been reported [41]. However, there are two other systems with the capability to image both the LL and PCTF simultaneously in vivo. Werkmeister et al. have obtained thickness of the LL based on measurements of OCT reflectance [42]. Using the principles of single thin film reflectometry, they presented the model of the OCT signal in a super-resolved regime. Rolland and colleagues also described an approach to calculate the thickness of the tear film and LL simultaneously [22]. Their system combined OCT imaging and statistical decision theory to estimate (not image) the thickness of the LL and aqueous layer. Although OCT is an effective method to measure the overall tear film, the accuracy of calculating LL thickness using statistical estimates or with OCT reflectivity both still needs further validation. Our current purpose-built optical system combines two established optical techniques that simultaneously measures the thickness of the LL and the overall tear film. These two techniques can function independent of each other, or can work together at a high degree of synchronization with specific co-registration of image locations for each system.

To demonstrate the capabilities of this multimodal system to simultaneously measure the thicknesses of both the PCTF and the LL in vivo, the effect of instilling artificial tears on the PCTF and PLTF was examined (with anticipated bulking). In both contact lens and non-contact lens wear, it could be observed from the OCT results that instillation of artificial tears increased the thickness of the overall tear film immediately, followed by a gradual reduction thereafter. These findings were consistent with other studies [22, 43–45]. However, unlike those previous reports, the thickness of the LL in this study was quantified simultaneously with the TDF subsystem (although bulking agents such as these artificial tears were not necessarily intended to increase the LL thickness). With the OCT subsystem, it is possible to spatially identify areas of the tear film that thin abnormally fast. At the same time, data from the TDF can show what is happening to the LL in those same areas.

Measurement of the tear film in vivo is difficult due to its dynamic nature. In a few seconds or less, the physical properties of the tear film (e.g. thickness, osmolarity, composition) can change due to evaporation, tearing, blinking, or drainage. To overcome this challenge, the TDF was equipped with a high-speed camera that images at 30 frames per second or higher if needed; this was found to be sufficient to capture and visualize the LL variations in this application. The current acquisition speed of the OCT only allowed sparse sampling of the ocular surface, which potentially resulted in a loss of image details. Strategies such as parallel excitation and detection in-line, termed line-field OCT, are currently being considered to obtain faster acquisition speeds [46, 47]. This would allow for a denser sampling of the ocular surface.

The imaging capabilities of the TDF and OCT were bound by optical limitations. The TDF system measured the thickness of the LL based on the dominant color of reflected light. The minimal detectable change in dominant color corresponded to a change of approximately 15 nm in LL thickness [48]. This was an inherent limitation of the TDF technique, which made it difficult to detect very subtle changes in LL thickness. The physical limitations of OCT imaging included dispersion, non-Gaussian spectral shape, and limited bandwidth of the light source. These factors together determined the minimal the axial resolution of the system (~ 1.38 µm). While this is sufficient for imaging a normal human PCTF (3–5 µm), we can expect the accuracy of thickness measurements to decrease as the PCTF evaporates and thins over time.

The field of view of the system was limited to a 3 mm diameter zone at the central cornea. This area allowed enough incident light to be reflected back to the detector. With a larger diameter zone, curvature of the cornea becomes a limiting factor. Light incident on the peripheral areas of a larger diameter zone would reflect off-axis and would not return to the instrument. Without light returning to the instrument (either to interfere with the reference arm, or to the TDF camera) thickness information would not be obtained. This issue could have been overcome using an objective lens with a larger aperture, however this type of lens was not readily available at the time. In addition, the spectral shaping method for OCT system needs further optimization to reduce the side lobe effect.

A geometrical measurement bias has to be considered in the thickness determination of both PCTF and LL. The thickness of PCTF measured via OCT is determined by the optical path length of the probe light through the layer of the tear film. When the probe beam is scanned away from the apex of the cornea, the light is no longer incident to the local surface and the optical path length through the tear film becomes longer than the actual thickness of the tear film, i.e. the thickness values measured via OCT appear larger than the true values. As reported from the simulations of dos Santos and colleagues, this bias is approximately 1% at 1.5 mm from the corneal apex and is not considered to significantly impact the results [43]. The TDF system illuminates and images the entire field at once, but the LLT of each pixel is still calculated via the optical path length of the probe beam through the lipid layer. Thus, the geometrical measurement bias influences the LLT values in a way similar to the OCT. Since the current study was limited to a zone of 1.5 mm radius at the central cornea, the geometrical biases were considered to be minute not to significantly impact the thickness measurements.

## Conclusion

In this work, we have described a novel imaging system that integrated OCT and TDF imaging techniques. The ability of precise measurement of the LL and overall tear film thickness may facilitate the study of many physiological and clinical aspects of the tear film.

## Abbreviations

PCTF: precorneal tear film
LL: lipid layer
OCT: optical coherence tomography
WDF: wavelength-dependent fringes
TDF: thickness-dependent fringes
ADF: angle-dependent fringes
FWHM: full width half maximum
GVD: group velocity dispersion
PSF: point spread function
PLTF: pre-lens tear film
